# Microbiota in cholestatic diseases: crosstalk among bile composition, the biliary microbiome, and host immunity

**DOI:** 10.3389/fimmu.2026.1884030

**Published:** 2026-07-22

**Authors:** Yanni Yang, Longfei Ren, Yulin Zhang, Xiaojuan Wang, Jin Shang, Lei Zhang

**Affiliations:** 1 The First School of Clinical Medicine, Lanzhou University, Lanzhou, China; 2 Department of Anesthesiology, The First Hospital of Lanzhou University, Lanzhou, Gansu, China; 3 Key Laboratory of Biotherapy and Regenerative Medicine of Gansu Province, The First Hospital of Lanzhou University, Lanzhou, China; 4 Department of General Surgery,The First Hospital of Lanzhou University, Lanzhou, China

**Keywords:** bile acid metabolism, bile acid-microbiome-immune axis, bile microbiome, cholestatic diseases, host immunity

## Abstract

Cholestatic liver diseases are a heterogeneous group of hepatobiliary disorders caused by impaired bile formation, secretion, or excretion, leading to hepatocyte injury, biliary inflammation, fibrosis, and eventually cirrhosis. Traditional studies have largely focused on isolated mechanisms, including bile acid toxicity, immune dysregulation, and genetic susceptibility. However, recent advances in metagenomics, metabolomics, and immunology have highlighted the critical role of the gut and biliary microbiota in disease pathogenesis. This review proposes the core concept of a “tripartite interplay among bile composition, biliary microbiome, and host immunity,” integrating the dynamic crosstalk among these three axes in cholestatic liver diseases. Bile composition shapes microbial communities and modulates immune responses through receptors such as FXR and TGR5. In turn, the biliary microbiome regulates bile acid metabolism and immune activity through microbial metabolites. Meanwhile, the host immune system senses microbial signals via pattern-recognition receptors, triggering inflammatory pathways and influencing microbial colonization and metabolism. These reciprocal interactions form complex feedback loops that drive disease progression from early inflammation to chronic fibrosis and cirrhosis. Based on this framework, emerging diagnostic approaches combine microbial signatures, bile acid profiles, and immune markers into multidimensional biomarker systems. Therapeutically, integrated strategies targeting the microbiome, bile acid metabolism, and immune pathways may offer synergistic benefits. Despite challenges including sampling difficulty, interindividual variability, and limitations of current models, future technologies such as single-cell sequencing, spatial transcriptomics, and multi-omics integration may enable precision diagnosis and targeted therapy.

## 1 Introduction

Cholestatic liver diseases, in a broad sense, refer to pathological conditions characterized by reduced bile flow or impaired excretion of bile constituents, leading to the accumulation of bile acids, bilirubin, and other biliary components in the liver and/or bloodstream (1, 2). Based on the site of obstruction, cholestasis can be classified into intrahepatic and extrahepatic cholestasis. Intrahepatic cholestasis primarily involves impaired bile synthesis or secretion at the hepatocyte level and disorders of the intrahepatic bile ducts. Common diseases include Primary Biliary Cholangitis (PBC), Primary Sclerosing Cholangitis (PSC), Progressive Familial Intrahepatic Cholestasis (PFIC), Alagille Syndrome (ALGS), and drug-induced liver injury (3). Among these, PBC is an autoimmune disease characterized by non-suppurative inflammation of the small and medium-sized bile ducts, whereas PSC is defined by diffuse inflammation, fibrosis, and structuring of the intrahepatic and/or extrahepatic bile ducts, and is frequently associated with Inflammatory Bowel Disease (IBD) (4, 5). Extrahepatic cholestasis is predominantly caused by mechanical obstruction of the biliary system, such as choledocholithiasis, biliary strictures, cholangiocarcinoma, compression by pancreatic cancer, and Biliary Atresia (BA), which is commonly observed in children (6, 7). The gut microbiota refers to the vast and diverse community of microorganisms colonizing the human gastrointestinal tract. The number of genes encoded by the gut microbiota far exceeds that of the human genome, earning it the designation of the “second genome” or “hidden organ” of the human body (8, 9). The number of bacteria colonizing the intestine of healthy adults is approximately 10^13^ to 10^14^, primarily comprising the phyla *Firmicutes*, *Bacteroidetes*, *Actinobacteria*, *Proteobacteria*, and *Verrucomicrobia*, with *Firmicutes* and *Bacteroidetes* typically constituting the dominant populations (10–12). The gut microbiota plays a central role not only in host nutrient absorption and metabolism but also, through dynamic interactions with the host immune system, participates in maintaining intestinal barrier integrity and defending against pathogen invasion, among other critical physiological functions (13, 14). Importantly, these diseases should not be regarded as a homogeneous group. PBC, PSC, BA, PFIC, and choledocholithiasis differ markedly in their initiating events, dominant pathological axes, microbiome alterations, bile acid profiles, and immune responses.

Traditional research perspectives on cholestatic liver diseases have often been limited to single factors or binary interactions, making it difficult to comprehensively elucidate their complex pathophysiological processes. Excessive accumulation of hydrophobic bile acids can induce hepatobiliary injury through detergent-like disruption of cell membranes, oxidative stress, mitochondrial dysfunction, and apoptosis (15, 16). Although this perspective highlights the importance of bile acids in disease progression, it fails to explain the significant interindividual variability in disease phenotype and prognosis observed under similar degrees of cholestasis. With advances in immunology, immune-mediated biliary injury has become a major research focus, particularly regarding the autoimmune mechanisms in PBC, leading to the identification of key evidence such as anti-mitochondrial antibodies (AMA) and T-cell infiltration (16–18). However, the limited efficacy of conventional immunosuppressive therapies in PSC suggests that disease progression is not driven solely by adaptive immunity, but also involves additional regulatory factors, including bile acid dysregulation, microbial imbalance, epithelial barrier dysfunction, and innate immune activation (19). The concept of the “tripartite interplay among bile composition–biliary microbiome–host immunity” has been proposed to overcome the limitations of traditional approaches. It emphasizes that bile composition (centered on bile acids, and also including cholesterol, phospholipids, immunoglobulins, etc.), the biliary microbiome (microbial communities colonizing the biliary system), and the host immune system (including both local biliary and systemic immunity) do not exist in isolation. Instead, they form an interdependent, mutually constraining, and dynamically balanced regulatory network through complex signal transduction and material exchange. Dysregulation within this network is considered a core driver in the pathogenesis and progression of cholestatic liver diseases (20, 21). By transcending the limitations of singular or binary perspectives, the concept of this tripartite interplay better reflects the multifactorial and complex nature of cholestatic liver diseases. In-depth investigation into this dynamic interactive network will enhance our understanding of the core disease mechanisms and open new research directions and therapeutic avenues for tackling this group of refractory disorders.

## 2 Characteristics of the bile microbiome and cholestatic diseases

### 2.1 Abnormal changes in bile composition during cholestasis

**Figure 1:**
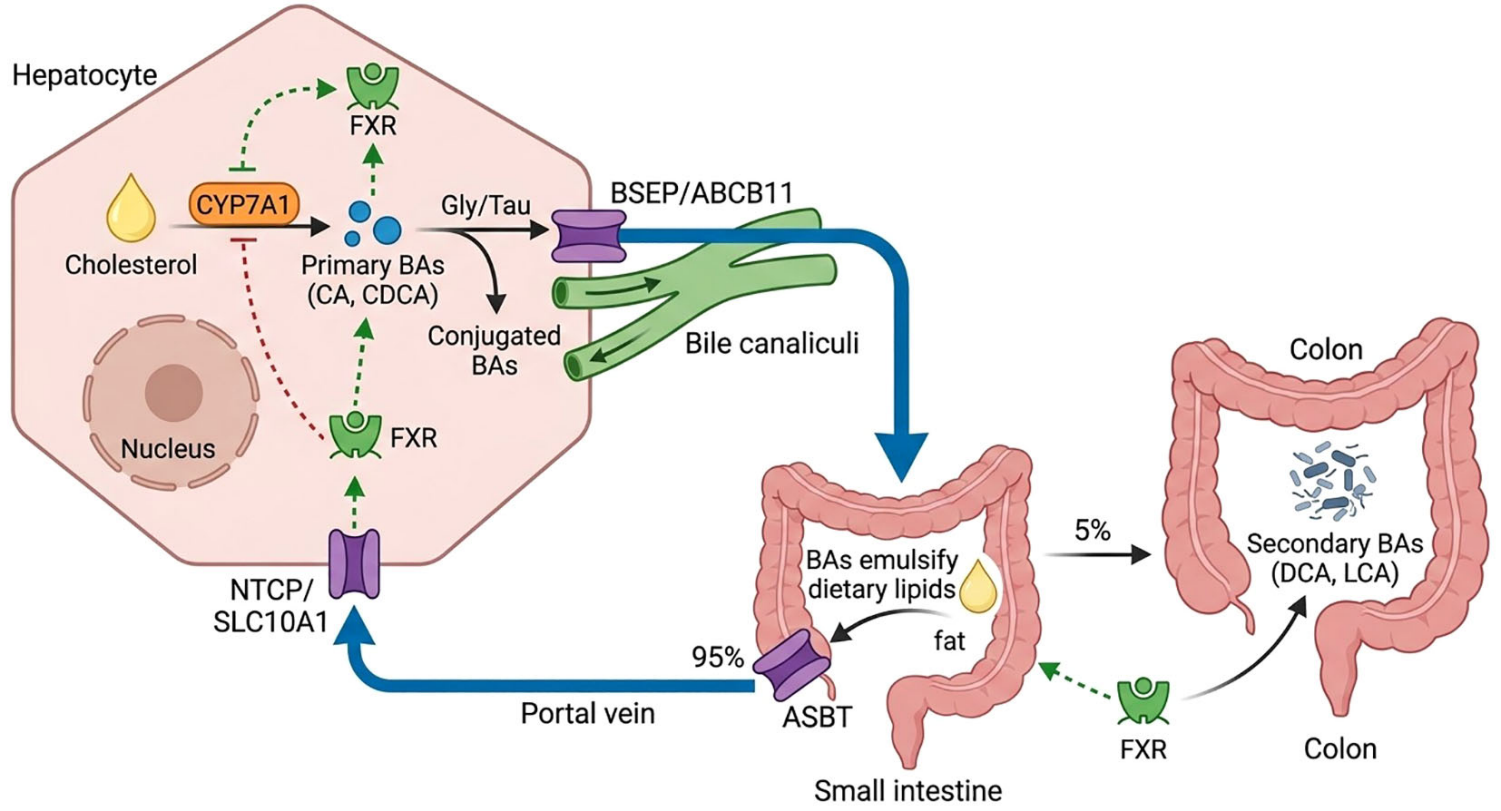
Enterohepatic circulation and FXR-mediated feedback control of bile acid homeostasis. In hepatocytes, cholesterol is converted to primary BAs via CYP7A1. Primary BAs are conjugated with glycine or taurine (Gly/Tau) and secreted into bile canaliculi through the bile salt export pump BSEP/ABCB11. After release into the small intestine, BAs emulsify dietary lipids and are efficiently reabsorbed (~95%) by the ASBT and returned to the liver via the portal vein, where they are taken up by NTCP/SLC10A1. A minor fraction (~5%) reaches the colon and is converted by the gut microbiota into secondary BAs (e.g., deoxycholic acid, DCA; lithocholic acid, LCA). FXR signaling in the liver and intestine provides negative-feedback regulation of BA synthesis, restraining CYP7A1 activity to maintain BA pool size and composition. ASBT, apical sodium-dependent bile acid transporter; BA, bile acid; BSEP, bile salt export pump; CDCA, chenodeoxycholic acid; CYP7A1, cholesterol 7α-hydroxylase; DCA, deoxycholic acid; FXR, farnesoid X receptor; Gly/Tau, glycine/taurine conjugation; LCA, lithocholic acid; NTCP, Na^+^-taurocholate cotransporting polypeptide; SLC10A1, solute carrier family 10 member 1; ABCB11, ATP-binding cassette subfamily B member 11.

Normal bile is composed of water, electrolytes, organic solutes (primarily including bile acids, cholesterol, and phospholipids), as well as small amounts of proteins, bilirubin, enzymes, and other bioactive substances. These components are synthesized and secreted in the liver, and their dynamic balance is maintained through a precisely regulated enterohepatic circulation. Together, they perform multiple physiological functions, such as digestion, absorption, metabolic regulation, and immune defense (22). Bile acids (BAs) are the most abundant organic components in bile and serve as the core effector molecules responsible for its physiological functions. In hepatocytes, BAs are synthesized from cholesterol through a series of enzymatic reactions, primarily catalyzed by cholesterol 7α-hydroxylase (CYP7A1), yielding primary bile acids such as cholic acid (CA) and chenodeoxycholic acid (CDCA). These are subsequently conjugated with glycine or taurine to form more water-soluble conjugated bile acids (23–25). Conjugated bile acids are actively secreted into the bile canaliculi via the bile salt export pump (BSEP/ABCB11), a process that constitutes the primary driving force for bile flow. They are then excreted into the small intestine, where they emulsify dietary lipids and facilitate the absorption of fat-soluble vitamins (26, 27). Approximately 95% of bile acids are actively reabsorbed in the terminal ileum by the apical sodium-dependent bile acid transporter (ASBT) and returned to the liver via the portal vein. They are then taken up by hepatocytes through the sodium–taurocholate cotransporting polypeptide (NTCP/SLC10A1) and resecreted into bile, forming an efficient enterohepatic circulation. Only about 5% of bile acids enter the colon, where they are converted by gut microbiota into secondary bile acids such as deoxycholic acid (DCA) and lithocholic acid (LCA) (28, 29). Throughout this process, bile acids activate receptors such as the hepatic and intestinal farnesoid X receptor (FXR), which in turn downregulates CYP7A1 expression and upregulates transporters including BSEP, thereby maintaining bile acid pool homeostasis and associated metabolic balance (30). **Figure 1** illustrates the tripartite interplay among bile composition, the biliary microbiome, and host immunity.

Cholestasis refers to a pathological condition characterized by impaired bile formation, obstructed bile flow, or inadequate bile excretion due to various etiologies, leading to the accumulation of bile components—particularly bile acids, bilirubin, and cholesterol—within the liver and their subsequent reflux into the systemic circulation (31, 32). This condition results in significant alterations in the composition, proportion, and concentration of bile constituents. These changes not only represent pathological manifestations of cholestasis but also constitute a critical pathophysiological basis for liver injury and disease progression. During cholestasis, the metabolism and excretion of bile acids are primarily impaired, resulting in their significant accumulation in the liver, blood, and urine. Concurrently, characteristic alterations occur in the composition and proportion of the bile acid pool (33, 34). During cholestasis, the total bile acid levels increase, accompanied by a series of pathologically significant alterations in their composition and proportions. Firstly, there is a predominant elevation of conjugated primary bile acids (particularly CA, CDCA, and their conjugated forms) in both serum and bile. This occurs due to impaired biliary excretion, which reduces intestinal bile acid levels, thereby diminishing the dehydroxylation and other transformations of primary bile acids by gut microbiota. As a result, the production of secondary bile acids (such as DCA and LCA) decreases, leading to an imbalance in the primary/secondary bile acid ratio (35, 36). In cholestatic diseases such as PBC and PSC, a decrease in secondary bile acids and an abnormal proportion of primary bile acids like CDCA have been consistently observed (37). Secondly, the ratio of hydrophobic to hydrophilic bile acids tends to shift unfavorably. Hydrophobic bile acids (e.g., CDCA, DCA, LCA) accumulate in the liver due to impaired excretion and defective transporter function, which constitutes a critical toxic mechanism underlying hepatocyte and cholangiocyte injury in hereditary cholestatic disorders such as PFIC. In contrast, exogenous administration of ursodeoxycholic acid (UDCA) increases the proportion of hydrophilic bile acids and dilutes hydrophobic components, thereby exerting cytoprotective effects. This represents a key mechanism of UDCA therapy in diseases like PBC (38–40). Furthermore, different bile acids exhibit varying agonist effects on receptors such as FXR and TGR5. The remodeling of the bile acid profile associated with cholestasis can lead to abnormal activation patterns of these receptors, subsequently disrupting cholesterol and glucose-lipid metabolism, as well as inflammatory and immune signaling pathways. These alterations contribute to the initiation and progression of liver injury in cholestasis (41, 42).

Under cholestatic conditions, the metabolism and secretion of cholesterol and phospholipids are markedly disrupted, leading to imbalances in their concentrations in bile and their ratios to bile acids. This exacerbates abnormalities in the physicochemical properties of bile and aggravates liver injury. During biliary obstruction or impaired hepatocellular secretory function, the excretion of bile acids and phospholipids decreases, whereas cholesterol secretion is relatively preserved or less reduced. Consequently, the cholesterol saturation index in bile increases, predisposing to the formation of cholesterol crystals and gallstones (43–45). Phospholipids, primarily phosphatidylcholine (PC), require transport into bile via MDR3/ABCB4. In hereditary cholestatic disorders such as progressive familial intrahepatic cholestasis type 3 (PFIC3) and acquired cholestatic diseases like PBC and PSC, MDR3 deficiency or downregulation of its expression/function leads to a significant reduction in biliary phospholipids. This not only impairs the solubilization of cholesterol but also diminishes the protective effect of phospholipids in binding to bile acids to neutralize the toxicity of hydrophobic bile acids (46, 47). Furthermore, intrahepatic accumulation of cholesterol and phospholipids can induce organelle stress and inflammatory signaling. Obstruction of biliary excretion also promotes their diversion into the bloodstream via alternative pathways, resulting in the formation of abnormal lipoproteins such as lipoprotein X (LP-X), which represents a characteristic biochemical feature of cholestatic liver disease (48, 49). **Table 1** summarizes the typical changes and pathophysiological significance of various bioactive constituents in bile during cholestasis, including bilirubin, biliary enzymes/proteins, inflammatory factors and chemoattractants, immune-related molecules, as well as cellular debris and oxidative stress products. The abnormalities of these constituents not only provide sensitive biochemical and immunological markers for cholestatic liver diseases, but also contribute to bile duct injury and the development and progression of liver fibrosis through multiple pathways, including amplification of inflammation, oxidative stress, and immune dysregulation.

**Table 1:** Alterations of other bioactive components in bile during cholestasis and their pathophysiological significance.

Category	Representative molecules	Typical changes in cholestasis	Major pathophysiological roles
Bilirubin	Conjugated and unconjugated bilirubin	Impaired biliary excretion and reflux into blood, causing conjugated hyperbilirubinemia and jaundice; reduced bilirubin in the intestinal lumen.	Long-term retention is toxic to hepatocytes and the central nervous system (e.g., kernicterus); decreased intestinal bilirubin may affect gut bacterial metabolism and contribute to dysbiosis.
Biliary enzymes/proteins	ALP, GGT, 5′-nucleotidase (5′-NT), etc.	Markedly increased activities in bile and serum, mainly released from injured cholangiocytes and canalicular membranes.	Sensitive biochemical markers of cholestasis and bile duct injury, widely used for diagnosis and assessment of disease severity.
Inflammatory mediators and chemokines	TNF-α, IL-6, IL-8, CCL2, CXCL10, etc.	Elevated levels in liver tissue, bile and serum.	Recruit and activate immune cells, amplify intrahepatic inflammation, and promote cholangiocyte damage and liver fibrogenesis.
Immune-related molecules	IgA, IgG, sIgA, complement, antimicrobial peptides (e.g., defensins), autoantibodies (e.g., AMA)	Increased biliary sIgA, complement and antimicrobial peptides as compensatory mucosal defense; AMA and other autoantibodies elevated in PBC.	Contribute to biliary mucosal defense but, when over-activated, also drive autoimmune-mediated cholangiocyte injury (e.g., in PBC).
Cellular debris and apoptotic bodies	Cellular fragments, apoptotic bodies, necrotic remnants	Accumulation in bile due to bile-acid- and inflammation-induced apoptosis/necrosis of hepatocytes and cholangiocytes.	Reflect ongoing cell injury and a deteriorating biliary microenvironment; further stimulate local inflammation and bile toxicity.
Oxidative stress–related products	ROS, lipid peroxides, etc.	Increased ROS and lipid peroxidation, largely driven by hydrophobic bile acids.	Damage membrane lipids, proteins and DNA of hepatocytes and cholangiocytes, thereby enhancing cell death, inflammation and fibrogenesis.

### 2.2 Imbalance of the bile microbiome in cholestatic diseases

For a long time, bile has been considered a relatively sterile environment, primarily due to the difficulty of detecting low-abundance or fastidious microorganisms using traditional culture methods (20). With the introduction of next-generation sequencing (NGS) technologies, particularly 16S rRNA gene sequencing, researchers have been able to conduct more comprehensive, culture-independent analyses of bacterial communities in bile samples. The positive detection rate and species resolution of these methods are generally superior to those of conventional culture (50, 51). Further application of whole-metagenome shotgun sequencing (WMS) not only enables more precise identification at the species or even strain level but also allows for the profiling of functional genes. This has revealed the potential roles of the bile microbiome in activities such as β-glucuronidase production, biofilm formation, and bile acid tolerance, providing clues for understanding gallstone formation and bile acid metabolism disorders (52, 53). However, the acquisition of bile samples still relies on invasive procedures such as endoscopic retrograde cholangiopancreatography (ERCP), percutaneous transhepatic cholangial drainage (PTCD), or surgery, which limits the availability of large sample sizes and healthy controls. Furthermore, low-biomass samples are susceptible to contamination from host DNA and environmental sources, imposing higher demands on DNA extraction protocols and bioinformatic processing (e.g., removal of background noise). Quantitative PCR is often employed to assess bacterial load and screen specimens suitable for metagenomic analysis (54). With improvements in sampling techniques and decreasing sequencing costs, the role of the bile microbiome in cholestatic diseases is expected to be elucidated in a more systematic and in-depth manner. Regarding the normal biliary microbiome, a unified definition is still lacking, primarily due to the difficulty in obtaining bile samples from truly healthy individuals. Existing data mostly come from liver transplant donors or patients without hepatobiliary diseases and are often confounded by factors such as hospitalization and antibiotic use (55, 56). Nevertheless, multiple studies have confirmed that bile under physiological conditions is not absolutely sterile but harbors a complex, albeit overall low-abundance, microbial community. The dominant phyla are typically *Firmicutes*, *Bacteroidetes*, *Actinobacteria*, and *Proteobacteria*. At the genus/family level, a higher abundance of commensal bacteria such as *Propionibacteriaceae* is observed, which is largely consistent with the microbial composition reported in the bile of non-stone patients (57). Overall, the alpha diversity of the normal biliary microbiome is low, which is considered an adaptation to the antimicrobial properties of bile acids and the relatively harsh environment within the biliary tract. However, the baseline for “normal” diversity still requires further definition through large-sample studies involving truly healthy populations. The compositional differences among various studies may be related to technical factors such as sample location (gallbladder bile vs. bile duct bile), sampling methods, and sequencing strategies. They also suggest that the sources of the biliary microbiome may be multifaceted: in addition to ascending migration from the gut, microbiota of oral/respiratory origin are more common in some bile samples, and hematogenous dissemination may also occur. The mechanisms underlying their colonization remain unclear (58, 59). Interestingly, microbial communities similar to the gut microbiota have also been found in the gallbladder of early vertebrates such as teleost fish, suggesting that the gallbladder microbiome and its interaction with host immunity may possess a certain degree of evolutionary conservation (60).

Cholestatic diseases are often accompanied by significant dysbiosis of the bile microbiome, characterized by disrupted microbial structure, altered diversity, and enrichment of specific pathogenic or opportunistic pathogens. Multiple studies have demonstrated significant differences in the alpha diversity (e.g., Shannon index, Chao1 index) of the bile microbiome between patients with cholestatic diseases and control groups, although the specific trends may vary depending on the disease type. In patients with primary choledocholithiasis (PC), all five measured alpha diversity indices of the bile microbiome were significantly reduced (61). The microbial diversity in the bile of patients with cholecystolithiasis was also lower than that in patients without stones (54). Conversely, in patients with common bile duct stones, the detected bacterial population diversity was higher than in patients without stones (6). These discrepancies may be related to factors such as sample selection, disease stage, the presence of co-existing infection, and detection methodologies. For instance, co-existing infection or inflammation may lead to the overgrowth of certain dominant bacteria, thereby reducing diversity; whereas during the process of stone formation, the colonization and proliferation of specific microbes might initially increase local diversity. The phylum *Proteobacteria*, as a major phylum of conditional pathogens, often predominates in patients with infectious cholestasis or cholelithiasis. For example, in patients with biliary obstruction, *Klebsiella pneumoniae*, *Acinetobacter baumannii*, and *Escherichia coli* are the most predominant bacteria (50). In the bile of patients with recurrent common bile duct stones, the abundance of *Proteobacteria* is also elevated, with the genus Klebsiella being dominant. *Escherichia-Shigella* is more common in patients with primary common bile duct stones and shows increased abundance in the duodenal microbiota of these patients (44, 62). Imbalance in the bile microbiome is not merely an accompanying phenomenon of cholestatic diseases; alterations in specific microbial communities may also correlate with disease severity, clinical prognosis, and the risk of complications. Multiple studies have demonstrated a close association between specific microbiota and the formation and recurrence of gallstones. In patients with primary common bile duct stones, the relative abundances of six bacterial taxa (*Actinobacteria* phylum, *Actinobacteria* class, Staphylococcales order, Micrococcales order, *Altererythrobacter*, and *Carnobacteriaceae*) were found to be associated with stone recurrence status (61). In patients with sphincter of Oddi laxity (SOL), a significant increase in the abundance of *Rhizobiaceae* was observed in the bile, and SOL was identified as an independent risk factor for common bile duct stone recurrence, with a shorter recurrence time. Furthermore, the abundance of the genus *Clostridium* in bile was significantly higher in the recurrence group compared to the non-recurrence group (63). These findings suggest that specific microbial communities may participate in the pathological processes of stone formation and recurrence by promoting calcium bilirubinate precipitation and cholesterol crystallization through the production of enzymes such as β-glucuronidase and phospholipase. For instance, β-glucuronidase-producing Streptococcus species (e.g., *Streptococcus dysgalactiae*, *Streptococcus agalactiae*) and *Parabacteroides merdae* (which harbors both *uidA* and *pldA* genes) are considered key bacterial species in the formation of pigment gallstones (64).

Beyond global shifts in microbial diversity, biliary dysbiosis should be interpreted at the species and functional levels. Several studies have suggested that biliary microbial communities in hepatobiliary diseases are frequently enriched with opportunistic pathogens, including *Enterococcus faecalis*, *Escherichia coli*, *Klebsiella pneumoniae*, *Enterobacter cloacae*, *Streptococcus* spp., *Pseudomonas aeruginosa*, and anaerobic taxa such as *Bacteroides* and *Clostridium* spp. These taxa are not merely passive colonizers but may exert disease-relevant functions. For example, Enterobacteriaceae such as *E. coli* and *K. pneumoniae* can provide lipopolysaccharide and other pathogen-associated molecular patterns that activate TLR4/NF-κB signaling, thereby amplifying epithelial inflammation and cholangiocyte injury (65–67). *Enterococcus* spp. may contribute to persistent biliary inflammation through biofilm formation, bile tolerance, and antibiotic resistance, which may partly explain recurrent cholangitis and incomplete microbial clearance after biliary drainage (68). Anaerobic bacteria with bile salt hydrolase or 7α-dehydroxylation activity may reshape the bile acid pool, thereby altering FXR- and TGR5-mediated metabolic and immune signaling (69). In addition, specific bacteria may promote epithelial barrier disruption, mucin remodeling, and inflammatory cytokine production, creating a permissive microenvironment for fibrosis, stone formation, and malignant transformation. To address the emerging complexity of the biliary ecosystem, increasing attention should also be paid to non-bacterial components, including the biliary virome and mycobiome (19, 70). Bacteriophages may influence biliary microbial ecology by regulating bacterial fitness, transferring virulence or antibiotic-resistance genes, and reshaping pathogen dominance under antibiotic or drainage pressure. Although evidence remains limited, fungal taxa such as *Candida* spp. may participate in biliary inflammation, particularly in immunocompromised patients or those undergoing repeated instrumentation. These fungal communities may interact with bacteria through biofilm formation and immune modulation. Therefore, future studies should move beyond 16S rRNA-based bacterial profiling and integrate metagenomics, metatranscriptomics, metabolomics, and culture-based validation to clarify how bacteria, bacteriophages, fungi, and host bile acid metabolism jointly shape biliary inflammation and disease progression.

## 3 Host immune mechanisms in cholestatic liver diseases

The biliary system, serving as a vital conduit connecting the liver and the intestine, is continuously exposed to microorganisms and their metabolites from the gut. Consequently, it has evolved a multi-layered immune defense network. This network employs innate immunity as the first line of defense, followed by adaptive immunity for specific responses, and is supported by a complex cytokine regulatory network, collectively maintaining the homeostasis of the biliary microenvironment. When this network is dysregulated, as seen in diseases such as PBC and PSC, aberrant activation and infiltration of immune cells directly lead to bile duct injury and the development of fibrosis.

### 3.1 Immune defense mechanisms of the biliary system

In cholestatic liver diseases, the innate and adaptive immune systems, along with the cytokine network, collectively constitute a critical “intermediary layer” underlying the imbalance of the hepatobiliary microenvironment. Innate immune cells serve as the first line of defense in biliary immunity, with Kupffer cells, neutrophils, and natural killer (NK) cells being particularly important. Kupffer cells recognize and clear pathogens and damage-associated molecular patterns (DAMPs) from the blood. However, in the context of bile acid accumulation, their activation pattern is altered. Bile acid accumulation also reshapes macrophage inflammatory responsiveness, linking cholestatic metabolic stress to impaired antimicrobial defense and inflammatory imbalance. While this may mitigate excessive inflammation, it can also potentially impair anti-infective capacity, which aligns with the clinical observation of increased susceptibility to infections in patients with cholestasis (71, 72). Neutrophils are heavily infiltrated in cholestatic models such as bile duct ligation (BDL). Their recruitment is closely associated with bile acid-induced mitochondrial dysfunction, ROS release, and NLRP3 inflammasome activation. The subsequent release of IL-1β and IL-18 further amplifies the inflammatory cascade (73, 74). Concurrently, myeloperoxidase (MPO) and matrix metalloproteinases (MMPs) released by neutrophils can directly damage biliary epithelial cells (BECs) and hepatocytes, promoting necrosis and fibrosis (15). NK cells mediate immune surveillance against infected or malignant BECs through perforin/granzyme and IFN-γ. In PBC, NK cells show prominent portal infiltration. Their cytotoxicity is modulated by bile acids; for instance, CDCA upregulates NKG2D expression and enhances the killing of cholangiocarcinoma cells, providing a clue for bile acid involvement in anti-tumor immunity (35, 75). Furthermore, group 3 innate lymphoid cells (ILC3s) promote biliary repair and maintain the intestinal barrier by producing IL-22. Abnormal bile acid profiles and microbial translocation can disrupt the RORγt–IL-22 axis, impairing biliary regenerative capacity (76, 77).

**Figure 2:**
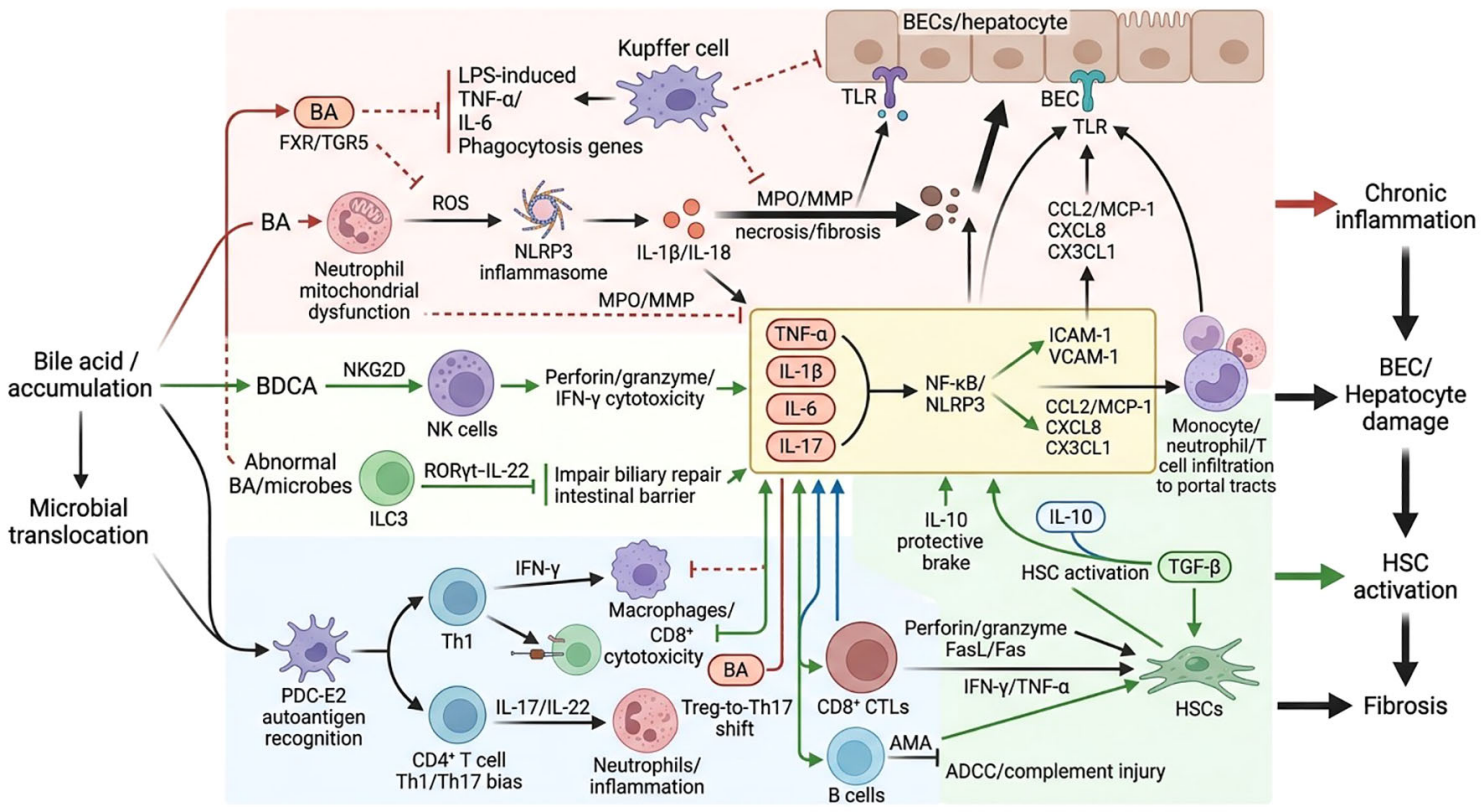
Immune–inflammatory crosstalk in cholestatic liver injury and fibrosis. Bile acid accumulation and microbial translocation activate Kupffer cells, neutrophils, NK cells, T cells, B cells, and biliary epithelial cells, leading to ROS production, NLRP3 inflammasome activation, cytokine release, chemokine-mediated immune-cell recruitment, and cytotoxic injury. Persistent inflammation promotes biliary epithelial/hepatocyte damage, hepatic stellate cell activation, and progressive fibrosis. BA, bile acid; BEC, biliary epithelial cell; HSC, hepatic stellate cell; ROS, reactive oxygen species; TLR, Toll-like receptor.

Adaptive immune cells play a decisive role in autoimmune cholestatic diseases. In diseases such as PBC and PSC, aberrant responses of CD4^+^ and CD8^+^ T cells against BECs form the core basis of bile duct destruction. Upon recognition of autoantigens such as pyruvate dehydrogenase complex-E2 (PDC-E2), CD4^+^ T cells exhibit a bias towards Th1/Th17 differentiation. Th1 cells secrete IFN-γ, promoting macrophage activation and enhancing the cytotoxicity of CD8^+^ T cells, while Th17 cells recruit neutrophils and amplify local inflammation via IL-17 and IL-22 (78, 79). In a bile acid-rich environment, the number and function of regulatory T cells (Tregs) are often suppressed. Certain bile acids can promote Treg polarization towards Th17 via STAT3 signaling, thereby breaking immune tolerance. In Mdr2^−/−^ mice, reducing intrahepatic bile acid levels restores Treg function and alleviates inflammation and fibrosis, highlighting the critical regulatory role of the bile acid–Treg–Th17 axis in disease progression (80). CD8^+^ T cells (cytotoxic T lymphocytes, CTLs) can directly induce apoptosis in BECs via the perforin/granzyme and FasL/Fas pathways and secrete IFN-γ and TNF-α to promote hepatic stellate cell (HSC) activation and fibrosis (81). B cells participate in bile duct-specific immune responses by producing AMA and functioning as antigen-presenting cells (APCs). They may directly mediate bile duct injury via complement and antibody-dependent cellular cytotoxicity (ADCC) and amplify T cell responses. In PSC, IgA-related abnormalities are also closely linked to intestinal barrier disruption and microbial translocation (82). As illustrated in **Figure 2**, cholestatic liver injury results from coordinated bile acid toxicity, microbial translocation, immune activation, and HSC-driven fibrogenesis.

### 3.2 Immune tolerance breakdown and inflammatory amplification

Beyond immune-cell infiltration, the progression of cholestatic liver diseases is characterized by a gradual breakdown of biliary immune tolerance. Under persistent cholestatic stress, biliary epithelial cells are not only passive targets of injury but also acquire immune-active properties, including enhanced cytokine and chemokine secretion and increased antigen-presenting capacity. This promotes the recruitment and activation of macrophages, dendritic cells, neutrophils, and lymphocytes within portal areas, thereby converting a protective immune response into chronic cholangitis (16, 82).

**Figure 3:**
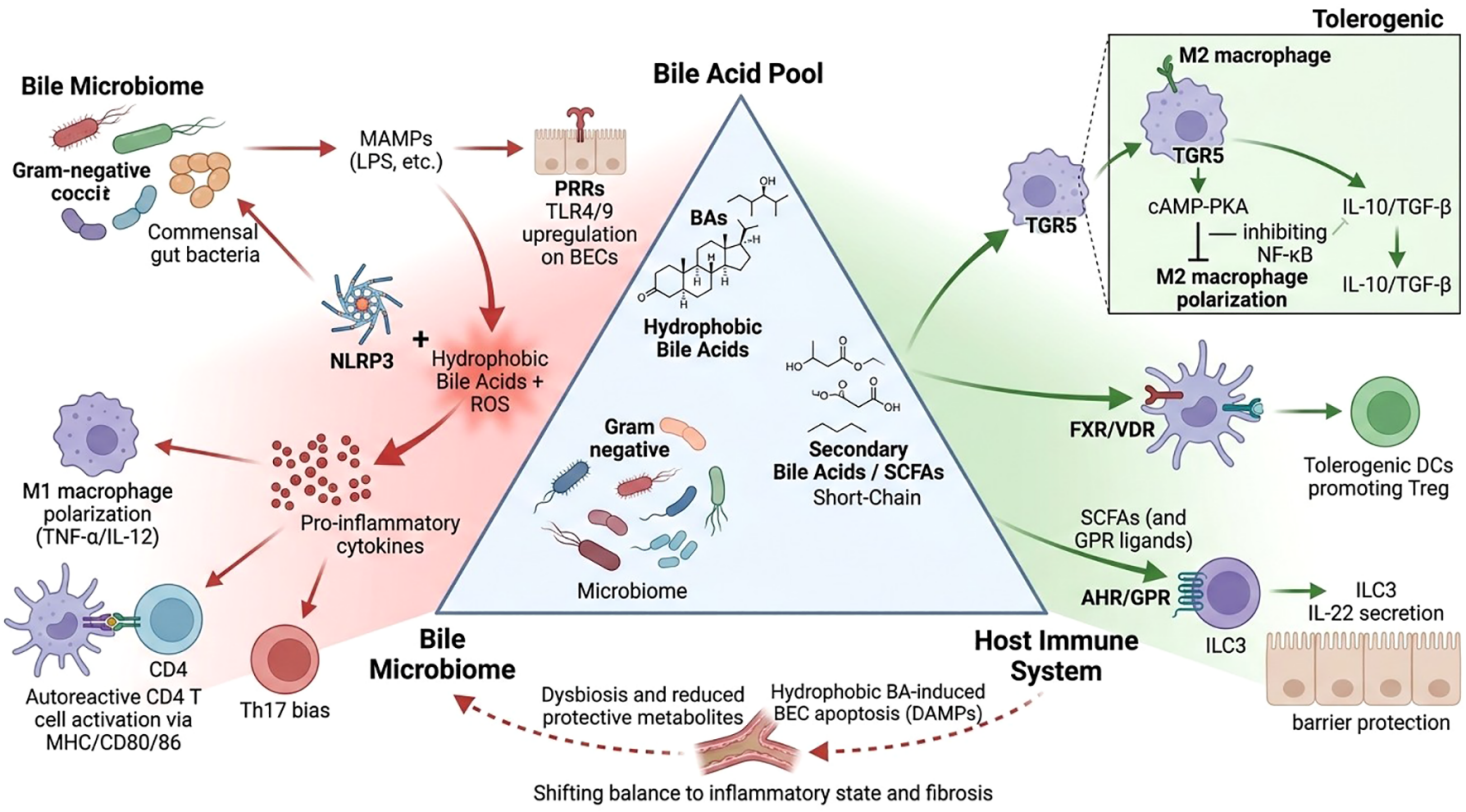
Crosstalk between the bile microbiome, bile acid pool, and host immune system shaping inflammatory versus tolerogenic responses. MAMPs (e.g., LPS) from biliary/commensal bacteria activate PRRs on BECs and upregulate TLR4/9. Together with hydrophobic BAs and ROS, this promotes NLRP3 inflammasome activation, triggering pro-inflammatory cytokine release, M1 polarization, autoreactive CD4^+^ T-cell activation, and Th17 skewing, thereby driving inflammation and fibrogenesis. In contrast, BA/metabolite-sensing pathways (TGR5, FXR/VDR, and SCFAs via AHR/GPR) support immune tolerance by promoting M2 macrophages, tolerogenic DC–Treg responses, ILC3-derived IL-22, and barrier protection. Dysbiosis and hydrophobic BA–induced BEC apoptosis (DAMPs) shift the balance toward a pro-inflammatory, profibrotic state. MAMPs, microbe-associated molecular patterns; LPS, lipopolysaccharide; PRRs, pattern-recognition receptors; BECs, biliary epithelial cells; TLR, Toll-like receptor; BAs, bile acids; ROS, reactive oxygen species; NLRP3, NOD-like receptor family pyrin domain-containing 3; TGR5, bile acid receptor (GPBAR1); FXR, farnesoid X receptor; VDR, vitamin D receptor; SCFAs, short-chain fatty acids; AHR, aryl hydrocarbon receptor; GPR, G-protein–coupled receptor; DC, dendritic cell; Treg, regulatory T cell; ILC3, group 3 innate lymphoid cell; IL, interleukin; DAMPs, damage-associated molecular patterns.

Adaptive immune dysregulation further amplifies this process. In autoimmune cholestatic diseases such as PBC and PSC, autoreactive CD4^+^ and CD8^+^ T cells, B-cell activation, autoantibody production, and impaired regulatory immunity collectively contribute to bile duct destruction (83, 84). In particular, an imbalance between pro-inflammatory Th17 responses and regulatory T-cell-mediated tolerance is closely associated with persistent inflammation, cholangiocyte injury, and fibrogenesis (85). Molecular mimicry between microbial antigens and biliary autoantigens may also participate in the initiation or perpetuation of autoimmune responses. Therefore, immune tolerance breakdown should be viewed as the downstream consequence of sustained inflammatory stimulation and antigenic exposure in the cholestatic microenvironment (86). As illustrated in **Figure 3**, bile microbiota communicates with the host immune system through two interconnected axes. On the one hand, microbiota-derived MAMPs activate PRRs and the NLRP3 inflammasome in biliary epithelial and hepatic innate immune cells, driving cytokine production and modulating the Treg versus effector T-cell balance. On the other hand, microbial remodeling of the bile acid pool acts on macrophages and dendritic cells via bile acid receptors to skew their polarization towards either inflammatory or tolerogenic phenotypes. The net outcome of these pathways determines whether the biliary immune milieu remains tolerant or progresses to chronic inflammation and autoimmune-mediated cholangiopathy in cholestatic liver disease. The upstream signals that drive this process, including microbiota-derived pattern-recognition receptor activation and bile acid receptor-mediated immune modulation, are discussed in detail in Sections 4.2 and 4.3 to avoid repetition.

## 4 A three-way interaction network between bile components, the bile microbiome, and host immunity

### 4.1 Interactions between bile components and the bile microbiome

Bile acids are the predominant organic components of bile and a key environmental factor shaping the biliary microbiome. Firstly, bile acids possess surfactant properties, enabling them to insert into and disrupt bacterial cell membranes, exerting concentration-dependent and species-specific bacteriostatic or bactericidal effects. Generally, Gram-positive bacteria are more susceptible, while some Gram-negative bacteria (e.g., certain E. coli strains) can acquire tolerance through mechanisms such as bile salt efflux pumps. Consequently, the concentration and hydrophobic/hydrophilic composition of bile acids determine the selective pressure on the biliary microbiome, which, under physiological conditions, helps maintain a steady-state microecology characterized by low bacterial load and low diversity in the biliary tract (87–89). Secondly, bile acids are also profound signaling molecules that can indirectly influence microbial community structure by modulating host barrier function and immunity. Activation of the FXR in intestinal and biliary epithelial cells by bile acids can induce the expression of antimicrobial peptides (e.g., RegIIIγ), thereby limiting the overgrowth of specific bacterial populations (90, 91). During cholestasis, impaired bile acid excretion leads to elevated levels in the liver and serum, while the total amount of bile acids entering the intestinal lumen and biliary tract decreases, accompanied by compositional imbalance (with a relative increase in hydrophobic components). This scenario, on one hand, enhances the inhibition of bile acid-sensitive bacteria and, on the other hand, provides a selective advantage for bile acid-tolerant bacteria and those harboring efflux pumps, ultimately reducing diversity and reshaping the community structure. This process is considered one of the significant triggers for dysbiosis associated with cholestatic diseases such as PBC and PSC (92, 93). Concurrently, ex vivo studies have shown that bile acids can regulate microbial metabolism and gene expression; for instance, they can induce bacteria to upregulate bile salt hydrolase (BSH) to detoxify and participate in reactions such as deconjugation and dehydroxylation of bile acids, further deepening the interdependence between bile acids and the microbiota. Conversely, the biliary microbiome is the primary executor of bile acid pool remodeling. Primary bile acids synthesized in the liver are secreted into bile in glycine- or taurine-conjugated forms. Approximately 95% are actively reabsorbed in the ileum via the ASBT and returned to the liver, while only about 5% escape reabsorption and enter the colon. There, they are transformed by the gut microbiota through deconjugation, 7α-dehydroxylation, and other processes into secondary bile acids, thereby altering the hydrophobicity, receptor activity, and toxicity of bile acids. Although the bacterial abundance within the bile lumen is relatively low, its metabolic capacity for bile acids is highly active. Particularly during cholestasis or biliary obstruction, more bacteria can ascend into the biliary tract and directly participate in local bile acid transformation, amplifying the positive feedback loop between bile acid pool dysregulation and biliary inflammation (94, 95). Because cholestatic liver diseases differ substantially in etiology, anatomical location, age of onset, immune background, and bile acid transport defects, the tripartite interplay among bile composition, the biliary microbiome, and host immunity should not be considered uniform across all disorders. Therefore, after discussing the three major interaction axes, we further compare disease-specific patterns of this network in major cholestatic diseases.

Microbial deconjugation, dehydroxylation, and other modifications of bile acids not only significantly enrich the chemical profile of bile acids but also alter their hydrophobicity and receptor activity, thereby differentially activating receptors such as FXR, TGR5, VDR, and RORγt. This influences bile acid synthesis and transport, glucose and lipid metabolism, and immune axes including Treg/Th17 (96, 97). In cholestatic liver diseases, intestinal dysbiosis leads to altered BSH and 7α-dehydroxylase activities, reduced secondary bile acids (DCA, LCA), and remodeling of the bile acid pool composition. This has been evidenced in patients with PBC and biliary atresia (98). Furthermore, a reduction in SCFA-producing bacteria and their metabolites can indirectly affect intrahepatic bile acid synthesis. In intrahepatic cholestasis of pregnancy (ICP) models, *Bacteroides* fragilis upregulates free bile acids via BSH activity, inhibits FXR signaling, and promotes excessive bile acid synthesis alongside impaired excretion, thereby further driving disease progression (99, 100). **Table 2** summarizes several key enzymatic reactions involved in the metabolic regulation of bile acids by the bile microbiome.

**Table 2:** Major bile acid–modifying reactions mediated by the bile microbiota.

Reaction type	Key enzymes/pathways	Representative taxa	Main products	Biological relevance
Deconjugation	Bile salt hydrolase	Lactobacillus, Bifidobacterium, Bacteroides, Clostridium	Conjugated CA/CDCA → free CA/CDCA	Reduces solubility and toxicity; provides substrates for further conversion
7α-dehydroxylation	7α-dehydroxylase complex	Clostridium scindens, C. hiranonis	CA → DCA; CDCA → LCA	Generates hydrophobic secondary bile acids with higher cytotoxicity
Epimerization	7α/7β-hydroxysteroid epimerase	Selected anaerobes (e.g. Clostridium spp.)	CDCA → UDCA	Produces UDCA with choleretic and cytoprotective effects
Oxidation/reduction	Hydroxysteroid dehydrogenases (HSDHs)	Various anaerobic bacteria	Oxo- and hydroxy–bile acid isomers	Alters hydrophobicity and receptor activity
Microbial conjugation (non-classical)	Bacterial amino acid conjugation	Bacteroides fragilis and others	Microbially conjugated bile acids (MBSCs)	Expands bile acid diversity; potential distinct immune/metabolic effects

### 4.2 Bidirectional regulation of the bile microbiome and host immunity

The bile microbiome and its metabolites serve as a critical nexus linking microbes to the biliary immune response through the interaction of MAMPs with host PRRs. MAMPs, including LPS from Gram-negative bacteria, peptidoglycan (PGN) and lipoteichoic acid (LTA) from Gram-positive bacteria, flagellin, viral double-stranded RNA, and fungal polysaccharides, are recognized by Toll-like receptors (TLRs), NOD-like receptors (NLRs), RIG-I-like receptors (RLRs), and C-type lectin receptors (CLRs) distributed on biliary epithelial cells, hepatocytes, macrophages, DCs, and neutrophils. This recognition initiates a series of innate immune signaling pathways (101, 102). During bile microbiome dysbiosis or ascending colonization of the biliary tract by gut-derived flora, axes such as LPS–TLR4 activate NF-κB and MAPK via MyD88/TRIF-dependent pathways, inducing the expression of TNF-α, IL-1β, IL-6, and chemoattractants such as CXCL8 and CCL2. This drives the recruitment of inflammatory cells and focal cholangitis. Viral/bacterial infections, intestinal barrier disruption, and bacterial translocation observed in patients with BA and PSC are believed to amplify hepatobiliary inflammation and fibrosis through similar mechanisms (103, 104).

The NLR family primarily senses intracellular bacterial components and cellular stress signals. Following recognition of bacterial peptidoglycan fragments, NOD2 activates NF-κB/MAPK, inducing the expression of antimicrobial peptides and pro-inflammatory factors. Genetic mutations in NOD2 are associated with increased susceptibility to Crohn’s disease and PSC, highlighting its importance in maintaining immune homeostasis of the gut–liver axis (105, 106). The NLRP3 inflammasome can be activated by various MAMPs, metabolites, and cholestasis-associated cellular damage signals, promoting caspase-1-mediated maturation and release of IL-1β and IL-18. It serves as a crucial molecular platform for inflammation amplification and hepatocyte injury in cholestatic liver diseases (107–109). Notably, low-level, persistent MAMPs–PRRs stimulation can also induce adaptive tolerance, promoting the production of IL-10 and TGF-β and the induction of Tregs. Therefore, this pathway is inherently a “double-edged sword,” with its net effect dependent on the intensity and duration of stimulation as well as the host’s immune context (110).

At the effector level, the bile microbiome first activates innate immune cells via the MAMPs–PRRs axis. Kupffer cells and infiltrating monocyte-derived macrophages recognize MAMPs, phagocytose pathogens, and secrete large amounts of TNF-α, IL-1β, IL-6, and CCL2, recruiting neutrophils and lymphocytes. In the context of bile acid accumulation, high concentrations of hydrophobic bile acids promote macrophage polarization toward a pro-inflammatory M1 phenotype, further exacerbating liver injury (111, 112). Driven by chemoattractants, neutrophils migrate into the bile ducts and hepatic sinusoids, clearing pathogens via phagocytosis, ROS, and neutrophil extracellular traps (NETs). However, excessive activation can lead to the release of proteases and inflammatory mediators, damaging biliary epithelial cells and hepatocytes (113). DCs act as bridging cells, taking up and processing microbial antigens for presentation to T cells. Their maturation status and cytokine profile can be reshaped by microbial metabolites such as SCFAs and secondary bile acids, determining whether pro-inflammatory Th1/Th17 cells or tolerogenic Tregs are induced (114–116).

**Figure 4:**
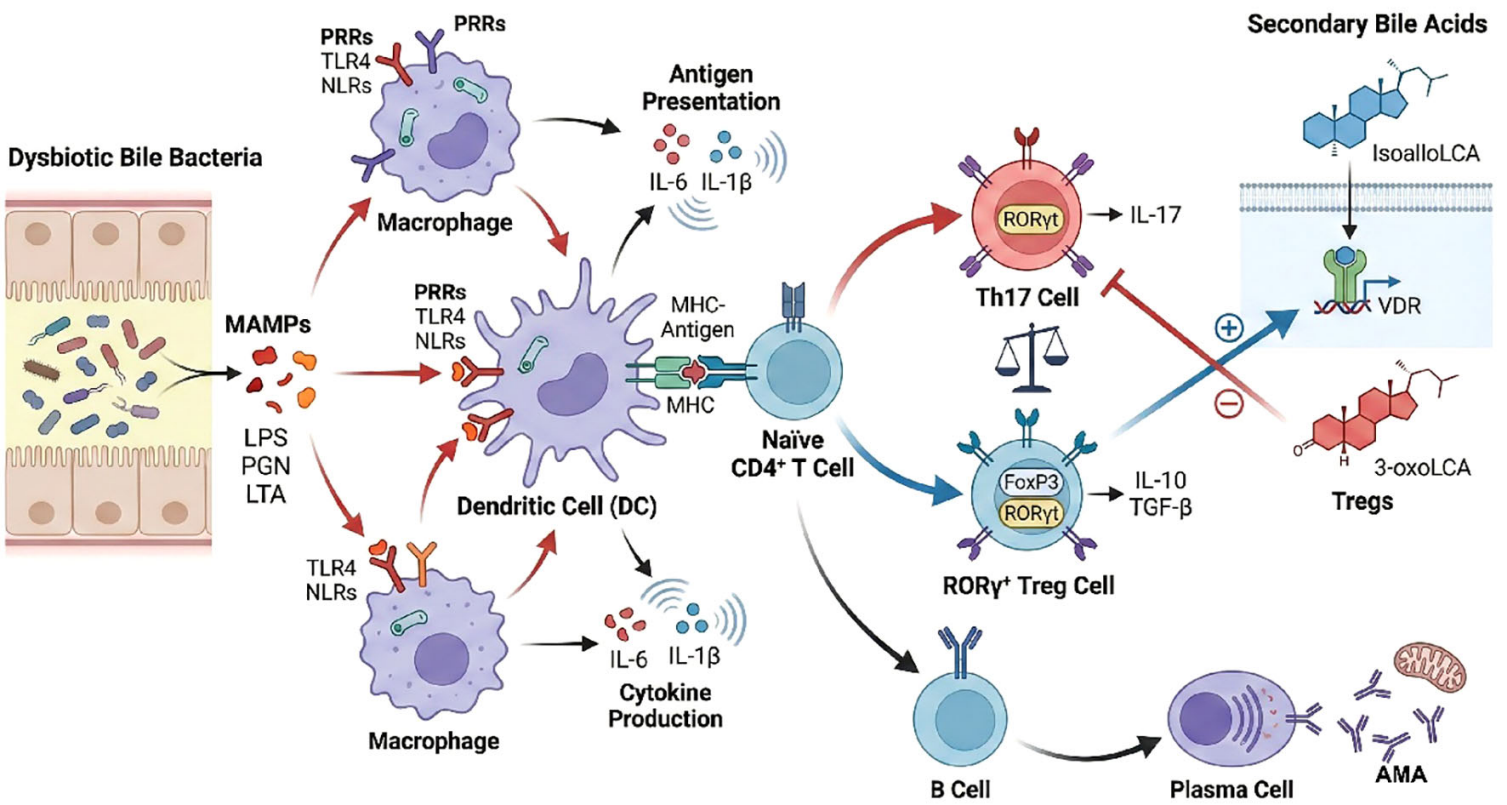
Dysbiotic bile microbiota–driven immune activation and bile acid–dependent Th17/Treg polarization. Microbial-associated molecular patterns (MAMPs; LPS, PGN, LTA) engage PRRs (TLR4, NLRs) on macrophages and dendritic cells, inducing IL-6/IL-1β and MHC-mediated antigen presentation to naïve CD4+ T cells, promoting Th17 (RORγt, IL-17) and RORγt+ Treg (FoxP3, IL-10/TGF-β) responses and downstream B-cell differentiation into plasma cells producing antimitochondrial autoantibodies (AMA). Secondary bile acids modulate this balance: isoalloLCA activates VDR to enhance Treg differentiation, whereas 3-oxoLCA inhibits Th17 polarization.

Adaptive immune responses unfold on the basis of innate immune activation and antigen presentation. DCs present microbial or cross-presented self-antigens to naïve CD4^+^ T cells via MHC II and co-stimulatory molecules, leading to their differentiation into Th1, Th2, Th17, or Tregs in specific cytokine milieus. In autoimmune cholestatic diseases such as PBC and PSC, an imbalance between Th17 and Tregs and impaired Treg function are commonly observed, which are closely linked to dysbiosis and abnormal bile acid profiles (117, 118). Stimulated by microbial antigens and helper T cells, B cells differentiate into plasma cells, producing autoantibodies including AMA. The gut microbiota promotes the activation of autoreactive B cells through molecular mimicry and tolerance breakdown, which is considered a key factor in the pathogenesis of PBC (119, 120). Overall, the bile microbiome links innate and adaptive immunity via the MAMPs–PRRs signaling axis, occupying a central position in the amplification of inflammation and the breakdown of immune tolerance in cholestatic diseases (121) (**Figure 4**).

### 4.3 Interactions between bile components and host immunity

Bile acids are a class of signaling molecules with “hormone-like” properties that finely regulate the differentiation, activation, and cytokine secretion of immune cells by activating nuclear receptors (FXR, PXR, VDR) and G protein-coupled receptors (TGR5/GPBAR1, S1PR2). These receptors are widely expressed on macrophages, DCs, T/B cells, and NK cells, enabling bile acids to directly act on multiple aspects of both innate and adaptive immunity (122, 123). Among them, FXR is the most central bile acid nuclear receptor. In addition to its high expression in the gut-liver axis, it is also present in macrophages, DCs, and certain T cell subsets. Activation of FXR can inhibit LPS-induced NF-κB/MAPK signaling, reduce the production of TNF-α, IL-1β, and IL-6, promote macrophage polarization towards an M2 phenotype, and induce tolerogenic DCs. It also suppresses effector T cell priming while upregulating tight junction proteins and antimicrobial peptides (e.g., RegIIIγ) to maintain intestinal barrier integrity (124–126). In cholestasis and ICP models, alterations in free bile acid levels by the BSH activity of gut microbiota (e.g., *Bacteroides fragilis*) can inhibit intestinal epithelial FXR signaling, impair antimicrobial peptide expression, and exacerbate inflammation. The partial efficacy of the FXR agonist obeticholic acid (OCA) in the treatment of PBC is believed to be related to the restoration of FXR signaling in both hepatocytes and immune cells (127). Secondary bile acid derivatives, such as isoallolithocholic acid (isoalloLCA), can promote the differentiation and homeostasis of RORγ^+^ Tregs by activating the vitamin D receptor (VDR). Conversely, 3-oxolithocholic acid (3-oxoLCA) restricts Th17 differentiation by inhibiting RORγt activity. This suggests that microbiota-dependent bile acid metabolism is a crucial upstream regulatory point for the Th17/Treg balance (128, 129).

TGR5 is another key bile acid receptor, expressed on macrophages, DCs, Tregs, as well as cholangiocytes and enteroendocrine cells, among others. Its activation inhibits NF-κB activity via the cAMP-PKA pathway, reduces the release of pro-inflammatory cytokines, and promotes the upregulation of IL-10 and downregulation of IL-12. This drives an immune response biased towards Th2 or Tregs, thereby enhancing immune tolerance (130–132). In intestinal L cells, TGR5 activation can also promote GLP-1 secretion, indirectly exerting anti-inflammatory effects (133). Conversely, aberrant TGR5 expression in cholangiocytes of patients with PSC is thought to be associated with biliary inflammation and fibrosis (134). VDR is also expressed in various immune cells. Some secondary bile acid derivatives, such as 3-oxoLCA and isoalloLCA, can act as its ligands: the former limits Th17 differentiation by inhibiting RORγt, while the latter promotes the differentiation of RORγ^+^ Tregs and enhances their suppressive function, thereby alleviating experimental colitis and maintaining mucosal tolerance (128).

**Figure 5:**
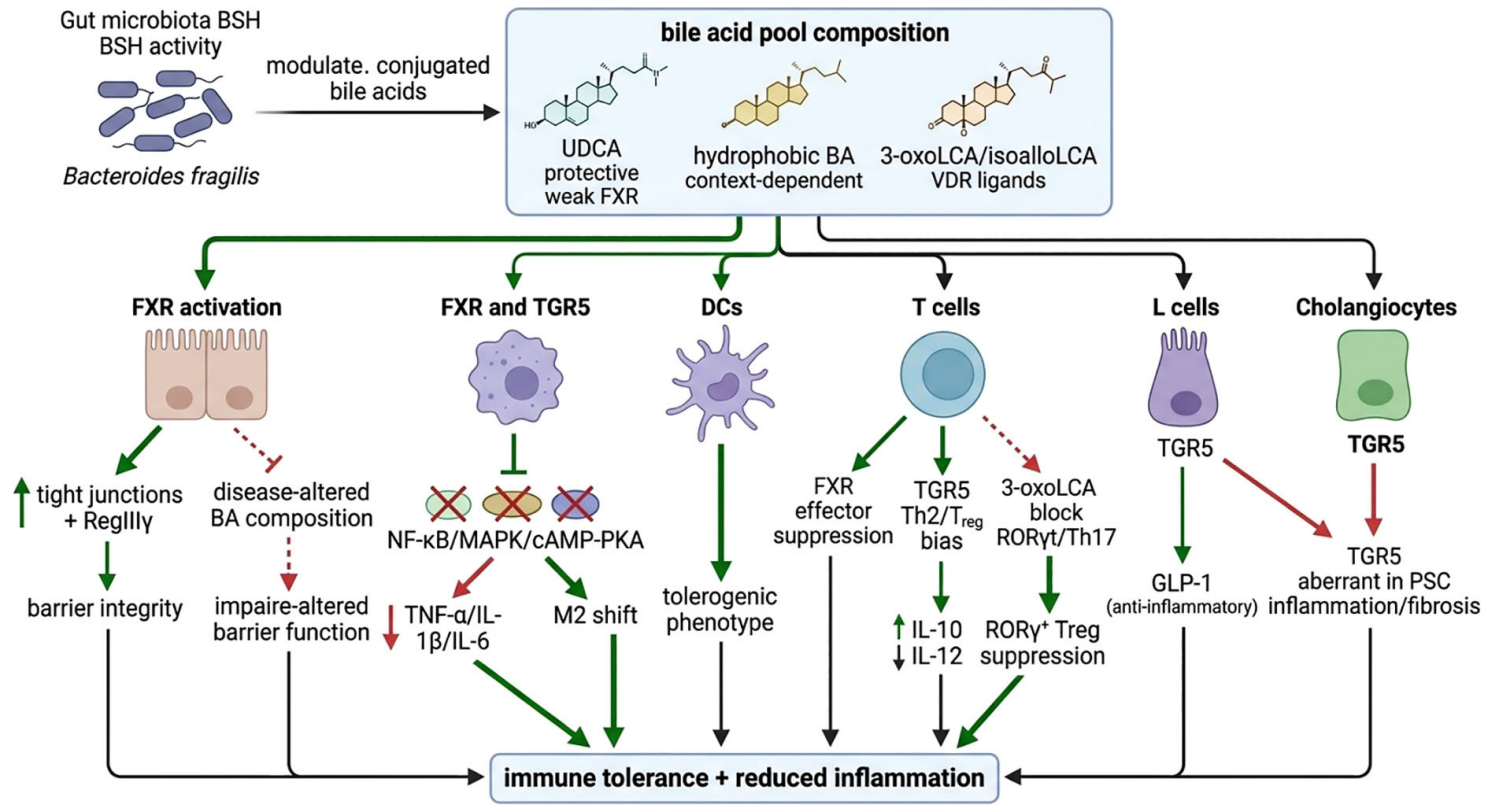
Microbiota–bile acid signaling in the regulation of intestinal and immune homeostasis. Gut microbial BSH activity reshapes the BA pool, generating BA species that regulate FXR-, TGR5-, and VDR-related pathways. These signals enhance epithelial barrier integrity, promote tolerogenic DCs and M2 macrophage polarization, modulate T-cell differentiation, stimulate GLP-1 release, and suppress pro-inflammatory signaling. In contrast, disease-altered BA composition may impair barrier function and promote cholangiocyte/PSC-related inflammation and fibrosis. BA, bile acid; BSH, bile salt hydrolase; DCs, dendritic cells; FXR, farnesoid X receptor; GLP-1, glucagon-like peptide-1; IL, interleukin; LCA, lithocholic acid; MAPK, mitogen-activated protein kinase; NF-κB, nuclear factor kappa B; PKA, protein kinase A; PSC, primary sclerosing cholangitis; RORγt, retinoic acid receptor-related orphan receptor γt; TGR5, Takeda G protein-coupled receptor 5; Tregs, regulatory T cells; UDCA, ursodeoxycholic acid; VDR, vitamin D receptor.

Furthermore, PXR can be activated by bile acids such as LCA, exerting anti-inflammatory effects at the gut-liver barrier by inhibiting NF-κB signaling. S1PR2 mediates the regulation of hepatocyte and immune cell migration and inflammatory responses by bile acids, although its immunological roles are still under investigation (92). Given the varying affinities and activation profiles of different bile acids for the aforementioned receptors, alterations in the composition of the bile acid pool (primary/secondary, hydrophobic/hydrophilic ratio) significantly influence the overall immune effects. The hydrophilic bile acid UDCA is a weak FXR activator but exerts protective effects through membrane stabilization, reduction of oxidative stress, and inhibition of inflammatory cytokine release. It has become a standard therapeutic agent for various cholestatic liver diseases. In contrast, more hydrophobic bile acids like LCA and DCA exhibit pro-inflammatory and cytotoxic effects at high concentrations. However, under specific doses and metabolic contexts, they can also produce anti-inflammatory and immunomodulatory effects via pathways such as VDR or TGR5 (135). As shown in **Figure 5**, gut microbiota-derived bile acid remodeling coordinates FXR-, TGR5-, and VDR-mediated signaling to maintain barrier integrity, immune tolerance, and inflammatory balance.

### 4.4 Disease-specific patterns of the tripartite interplay in major cholestatic disorders

Although the tripartite framework of bile composition, the biliary microbiome, and host immunity provides a useful integrative model, its relative contribution differs across cholestatic disorders. In PBC, the dominant feature is autoimmune-mediated destruction of small intrahepatic bile ducts. Therefore, the host immune axis is particularly prominent, characterized by antimitochondrial antibodies, autoreactive T-cell responses, B-cell activation, and progressive loss of immune tolerance toward biliary epithelial cells. Microbial dysbiosis and altered bile acid metabolism may contribute to this process by promoting molecular mimicry, changing antigen exposure, and reshaping regulatory immune responses. In this context, the bile acid axis is clinically relevant not only because cholestasis modifies the hydrophobic/hydrophilic bile acid balance, but also because therapeutic agents such as UDCA and FXR agonists may partially restore bile acid homeostasis and immune regulation. PSC displays a different pattern, in which the gut-liver axis is more central. The frequent coexistence of PSC with IBD suggests that intestinal barrier disruption, microbial translocation, and portal delivery of microbial products are key upstream drivers of hepatobiliary inflammation. Compared with PBC, PSC is less clearly dominated by classical autoimmunity and is more strongly associated with chronic cholangitis, periductal fibrosis, and persistent activation of innate immune pathways. Dysbiosis-related MAMPs may activate TLR/NLR signaling in biliary epithelial cells, Kupffer cells, and dendritic cells, thereby promoting cytokine release, antigen presentation, and fibrogenesis. In addition, altered bile acid profiles and reduced production of protective secondary bile acids may weaken epithelial barrier function and impair tolerogenic immune responses. Thus, PSC can be regarded as a prototypical disease in which intestinal dysbiosis, bile acid remodeling, and immune activation form a self-amplifying gut–liver–bile duct inflammatory loop.

Biliary atresia represents a pediatric cholestatic disorder with distinct developmental and inflammatory features. Unlike adult autoimmune cholangiopathies, BA is characterized by early-life fibroinflammatory obstruction of the extrahepatic bile ducts. Viral infection, abnormal innate immune activation, and age-dependent immaturity of the gut and biliary microbiome may contribute to disease initiation and progression. In this setting, reduced bile flow can rapidly alter intestinal microbial colonization, decrease luminal bile acid delivery, and promote dysbiosis. Conversely, microbial products may further stimulate innate immune responses and accelerate bile duct injury and fibrosis. Therefore, the tripartite network in BA may be dominated by early-life microbiome development, innate immune activation, and progressive obstruction-driven bile acid retention. PFIC differs from PBC, PSC, and BA in that the primary abnormality is usually genetic impairment of bile formation or transport. Defects in transporters such as BSEP or MDR3 directly disturb bile acid secretion or phospholipid-dependent protection against bile acid toxicity. Consequently, the bile composition axis is the initial driver of injury. The accumulation of toxic bile acids causes hepatocellular and cholangiocellular stress, which secondarily induces inflammatory signaling and fibrosis. Microbiome changes may occur as a consequence of altered bile acid delivery to the intestine and modified antimicrobial pressure, but they are less likely to represent the primary initiating factor. Therefore, in PFIC, therapeutic strategies should focus primarily on correcting bile acid transport, reducing bile acid toxicity, or interrupting enterohepatic circulation, while microbiome- and immune-targeted approaches may serve as adjunctive interventions. Choledocholithiasis and related obstructive cholestatic disorders show another disease-specific pattern, in which local biliary colonization and microbial enzymatic activity are particularly relevant. Biliary obstruction changes bile flow dynamics and creates a permissive niche for bacterial colonization, biofilm formation, and recurrent infection. Specific microorganisms may promote pigment stone formation through β-glucuronidase activity and contribute to cholesterol crystallization through bile acid deconjugation and phospholipid modification. Compared with PBC or PSC, immune activation in choledocholithiasis is more often secondary to obstruction, infection, and cholangitis rather than primary immune dysregulation. Therefore, the tripartite interplay in choledocholithiasis is dominated by obstruction-induced bile stasis, microbial colonization, and local inflammatory amplification. Taken together, PBC may be viewed as an immune-dominant autoimmune cholangiopathy, PSC as a gut-liver axis-dominant inflammatory cholangiopathy, BA as an early-life fibroinflammatory obstructive cholangiopathy, PFIC as a bile composition/transport-dominant genetic cholestasis, and choledocholithiasis as an obstruction- and infection-associated biliary microbiome disorder. Recognizing these disease-specific patterns is essential for developing precision diagnostic and therapeutic strategies based on microbial signatures, bile acid profiles, and immune biomarkers.

The PSC–IBD phenotype deserves particular attention because it provides one of the clearest clinical examples of the gut–liver axis in cholestatic disease. In PSC-IBD, intestinal inflammation and barrier dysfunction facilitate the translocation of microbial products into the portal circulation, exposing the liver and biliary tree to persistent MAMP stimulation. This may activate TLR/NLR signaling in Kupffer cells, dendritic cells, and biliary epithelial cells, leading to cytokine production, antigen presentation, and recruitment of inflammatory lymphocytes. Meanwhile, intestinal dysbiosis alters bile acid transformation, reducing protective secondary bile acids and weakening bile acid receptor-mediated barrier and immune regulation. These mechanisms may explain why PSC is closely linked to chronic cholangitis, periductal fibrosis, and poor response to conventional immunosuppression. Therefore, PSC should be discussed not simply as another cholestatic disorder, but as a distinct gut–liver axis-driven disease model.

## 5 Therapeutic implications of targeting the bile acid–biliary microbiome–immune network

The bile acid–biliary microbiome–immune network provides several potentially druggable nodes for hepatobiliary diseases. Rather than targeting a single pathological event, therapeutic strategies may be more effective when they are designed to restore bile acid homeostasis, reshape microbial ecology, and attenuate immune-mediated biliary injury simultaneously.

Bile acid receptors represent central regulatory nodes linking bile acid metabolism to inflammation and epithelial homeostasis. FXR activation can reduce bile acid synthesis, promote bile acid transport, improve epithelial barrier integrity, and suppress inflammatory signaling. Therefore, FXR agonists may be particularly relevant in cholestatic and inflammatory biliary diseases characterized by excessive bile acid accumulation and immune activation (136). TGR5 agonism may also exert anti-inflammatory effects by regulating macrophage activation, cytokine production, and bile acid-induced immune responses. However, the therapeutic application of FXR/TGR5 agonists remains context-dependent, as excessive or inappropriate bile acid receptor activation may have different consequences according to disease stage, bile acid composition, and microbial metabolic activity (137, 138). Future studies should clarify whether combined modulation of FXR/TGR5 signaling and biliary microbiota can achieve more durable control of biliary inflammation.

Antibiotics remain clinically important for controlling acute bacterial cholangitis and biliary infections, especially when opportunistic pathogens such as *Enterococcus*, *Escherichia coli*, *Klebsiella*, and *Pseudomonas* dominate the biliary ecosystem (139, 140). Beyond infection control, antibiotics may transiently reduce microbial-derived pathogen-associated molecular patterns, including lipopolysaccharide, thereby attenuating TLR4/NF-κB-driven inflammation. However, repeated or broad-spectrum antibiotic exposure may also aggravate dysbiosis, select multidrug-resistant bacteria, disrupt bile acid-transforming commensals, and promote recurrent infection or biofilm persistence (68, 140). Therefore, future antimicrobial strategies should shift from empirical broad-spectrum suppression toward precision microbiome-guided therapy, including pathogen-specific antibiotics, biofilm-disrupting agents, antimicrobial peptides, and possibly bacteriophage-based approaches (68, 70). Probiotics and prebiotics may offer a safer strategy to restore microbial balance and reinforce the gut–liver–biliary axis. Potential mechanisms include increasing colonization resistance against pathobionts, improving epithelial barrier function, modulating bile salt hydrolase activity, and regulating bile acid-derived immune signaling (141). Microbial metabolite-based interventions, such as short-chain fatty acids or secondary bile acid-related modulation, may also affect macrophage polarization, Treg differentiation, and inflammatory cytokine production (142). Nevertheless, evidence in biliary diseases remains less mature than in intestinal diseases, and strain specificity is a major limitation. Future studies should identify which probiotic strains or microbial metabolites can reproducibly modify bile acid profiles and immune responses in specific hepatobiliary disease settings.

FMT represents an ecosystem-level strategy for correcting microbial dysbiosis. In primary sclerosing cholangitis, early clinical evidence suggests that FMT is feasible and may increase microbial diversity, although its efficacy remains preliminary and requires validation in larger controlled trials. FMT may influence the biliary disease network by restoring gut microbial diversity, altering bile acid transformation, reducing pathobiont expansion, and indirectly modulating hepatic and biliary immune responses (143). However, its use in hepatobiliary diseases should be cautious because many patients have impaired barrier function, cholestasis, recurrent infection, or immunosuppression. Donor selection, engraftment assessment, long-term safety, and standardized endpoints remain essential challenges before FMT can be routinely recommended (144). Ileal bile acid transporter inhibitors reduce intestinal bile acid reabsorption and interrupt enterohepatic circulation, thereby lowering systemic and hepatic bile acid burden. This strategy is clinically attractive for cholestatic disorders, particularly those associated with pruritus and bile acid accumulation (145). By decreasing bile acid overload, IBAT inhibitors may indirectly reduce bile acid-induced epithelial injury, inflammatory activation, and microbial ecological pressure. However, their influence on the biliary microbiome and immune microenvironment remains insufficiently defined. Future studies should evaluate whether IBAT inhibition can reshape bile acid pools, microbial metabolism, and biliary inflammation in an integrated manner.

Given the reciprocal interactions among bile acids, biliary microbiota, and immune responses, single-target therapy may be insufficient for complex hepatobiliary diseases. A future precision strategy may combine bile acid receptor modulators, microbiome-directed interventions, and anti-inflammatory therapies according to disease stage and molecular phenotype. For example, patients dominated by pathogenic Enterobacteriaceae may benefit from microbiome-guided antimicrobial or phage-based approaches, whereas patients with bile acid accumulation and receptor dysregulation may be more suitable for FXR/TGR5 modulation or IBAT inhibition (70). Integration of metagenomics, bile acid metabolomics, inflammatory biomarkers, and clinical phenotyping will be essential for identifying responsive patient subgroups and optimizing therapeutic windows (136, 146).

## 6 Future directions and challenges

Although the theoretical framework of the “bile composition–biliary microbiome–host immunity” tripartite interplay provides novel insights into the diagnosis and treatment of cholestatic liver diseases, translating basic research findings into clinical applications still faces numerous challenges. Difficulties in sample acquisition, the complexity of individual variations, and the heterogeneity of the diseases themselves constitute major bottlenecks that severely hinder the clinical translation of novel diagnostic biomarkers and therapeutic strategies.

The acquisition of bile and biliary tissue samples is crucial for studying the biliary microbiome and the local immune microenvironment, yet it encounters significant ethical and technical obstacles in clinical practice. Bile collection typically requires invasive procedures such as ERCP, PTCD, or surgery. While these methods allow direct access to bile, they carry risks of complications including bleeding, infection, and biliary injury. These risks are notably increased, particularly in critically ill patients or those with concomitant cirrhosis (147). For instance, in patients with PSC, ERCP is used not only for diagnosis but also for the management of biliary strictures; however, approximately 5%–10% of patients experience post-procedural complications such as pancreatitis or cholangitis (148). Consequently, it is clinically challenging to routinely obtain bile samples for research purposes, leading to a scarcity of large-scale study data on the biliary microbiome and bile composition. This scarcity limits the discovery and validation of related diagnostic biomarkers. Furthermore, bile samples are susceptible to contamination by skin and intestinal flora during collection, transportation, and processing. Strict quality control to exclude contaminant interference is therefore a prerequisite for ensuring the reliability of research findings. Although some non-invasive or minimally invasive sampling techniques have been developed in recent years, such as duodenal fluid aspiration and capsule endoscopy sampling, the feasibility and sample representativeness of these methods require further validation (149).

Individual variation represents another critical factor affecting clinical translation, manifesting primarily across multiple dimensions including the gut/biliary microbiome, bile acid metabolic profiles, immune response status, and genetic background. The composition of the gut and biliary microbiomes is influenced by a multitude of factors such as age, sex, diet, geography, lifestyle habits, and medication use (particularly antibiotics), resulting in substantial inter-individual differences (150). For example, the characteristics of gut dysbiosis in patients with PBC show significant variations across different ethnicities and regions. In European and American populations, a reduction in *Bacteroidetes* and an increase in *Firmicutes* are commonly observed, whereas Asian populations may exhibit abnormal changes in *Actinobacteria (*151). Such individual variability in microbial composition makes it difficult to standardize microbiome-based diagnostic biomarkers and therapeutic strategies (e.g., probiotics, fecal microbiota transplantation), leading to considerable fluctuation in clinical efficacy. Bile acid metabolic profiles also exhibit significant individual differences. Even in healthy individuals, the size of the bile acid pool and the proportional composition of bile acids undergo physiological fluctuations. In patients with cholestatic diseases, abnormal alterations in the bile acid profile are further complicated by factors such as genetic background, disease stage, and therapeutic drugs (152). For instance, patients with PFIC3 carrying ABCB4 gene mutations, compared to those with BSEP deficiency, show significantly lower phospholipid levels in bile, leading to stronger toxic effects of bile acids and potentially different responses to ileal bile acid transporter (IBAT) inhibitor therapy (153).

Based on the current understanding of the “bile composition–biliary microbiome–host immunity” tripartite interplay in cholestatic diseases and the limitations of existing research, future studies should focus on several concrete priorities. First, the core mechanisms underlying this tripartite interaction should be further clarified through standardized bile sampling and microbiome analytical protocols, as biliary microbial profiles may be affected by sampling site, ERCP manipulation, prior antibiotic exposure, stent placement, storage conditions, sequencing depth, and contamination control. Second, future studies should avoid treating cholestatic liver diseases as a single entity and should instead establish disease-specific multi-omics cohorts integrating bile acid profiling, biliary/gut microbiome analysis, metagenomics or metatranscriptomics, and immune phenotyping, thereby moving beyond descriptive dysbiosis toward functional interpretation. Third, more physiologically relevant experimental models, including humanized or gnotobiotic mouse models colonized with patient-derived microbiota, are needed to establish causal links among bile acid transformation, FXR/TGR5 signaling, microbial imbalance, and immune activation. Finally, clinical studies and trials evaluating microbiome- or bile acid-targeted interventions should incorporate longitudinal microbiome profiling, bile acid measurements, inflammatory biomarkers, and safety monitoring to support individualized diagnosis and treatment systems, precise modulation of the tripartite balance, and new strategies for disease prevention. Through interdisciplinary integration and technological innovation, these efforts may advance this field from mechanistic discovery toward clinical application.
